# The class I-specific HDAC inhibitor MS-275 modulates the differentiation potential of mouse embryonic stem cells

**DOI:** 10.1242/bio.20135587

**Published:** 2013-08-22

**Authors:** Gianluigi Franci, Laura Casalino, Francesca Petraglia, Marco Miceli, Roberta Menafra, Branka Radic, Valeria Tarallo, Monica Vitale, Marzia Scarfò, Gabriella Pocsfalvi, Alfonso Baldi, Concetta Ambrosino, Nicola Zambrano, Eduardo Patriarca, Sandro De Falco, Gabriella Minchiotti, Hendrik G. Stunnenberg, Lucia Altucci

**Affiliations:** 1 Dipartimento di Biochimica, Biofisica e Patologia Generale, Seconda Università degli Studi di Napoli, Vico L. De Crecchio 7, 80138 Napoli, Italy; 2 Department of Molecular Biology, Faculties of Science and Medicine, Radboud University, Nijmegen Center for Molecular Life Sciences, 6500 HB Nijmegen, The Netherlands; 3 Institute of Genetics and Biophysics “A. Buzzati-Traverso”, Stem Cell Fate Laboratory, National Research Council, 80131 Napoli, Italy; 4 CEINGE, Via G. Salvatore 486, 80125 Napoli, Italy; 5 Dipartimento di Medicina Molecolare e Biotecnologie Mediche, Università di Napoli Federico II, 80131 Napoli, Italy; 6 IRGS, Biogem, 83031 Ariano Irpino, Avellino, Italy; 7 Institute of Protein Biochemistry–CNR, Via P. Castellino 111, 80131 Napoli, Italy; 8 Department of Environmental and Life Science, Second University of Naples, 81100 Caserta, Italy

**Keywords:** Stem cell, Epigenetic, HDACi

## Abstract

Exploitation of embryonic stem cells (ESC) for therapeutic use and biomedical applications is severely hampered by the risk of teratocarcinoma formation. Here, we performed a screen of selected epi-modulating compounds and demonstrate that a transient exposure of mouse ESC to MS-275 (Entinostat), a class I histone deacetylase inhibitor (HDAC), modulates differentiation and prevents teratocarcinoma formation. Morphological and molecular data indicate that MS-275-primed ESCs are committed towards neural differentiation, which is supported by transcriptome analyses. Interestingly, *in vitro* withdrawal of MS-275 reverses the primed cells to the pluripotent state. *In vivo*, MS275-primed ES cells injected into recipient mice give only rise to benign teratomas but not teratocarcinomas with prevalence of neural-derived structures. In agreement, MS-275-primed ESC are unable to colonize blastocysts. These findings provide evidence that a transient alteration of acetylation alters the ESC fate.

## Introduction

Epigenetic regulation refers to the modulation of gene expression in cellular heritability, fate, development and (re)programming other than through alterations of the DNA sequence itself (Kim et al., 2010; Orkin and Hochedlinger, 2011). Aberrant epigenetic regulation has been directly linked to human diseases such as cancer (Martens et al., 2010) and epigenetic modulating compounds, the so-called ‘epidrugs’, have entered into the clinic (Manzo et al., 2009). A plethora of studies have revealed that in embryonic stem cells (ESCs) the structure of chromatin influences the expression of the pluripotency genes and characteristics, such as the transcription factors (TFs) Oct4 and Nanog (Chambers et al., 2003; Loh et al., 2006; Niwa et al., 2000). The relationship between TFs and chromatin organization is essential for both stem cell potential and differentiation (Arney and Fisher, 2004; Atkinson and Armstrong, 2008; Bernstein et al., 2006; Dovey et al., 2010; Golob et al., 2008; Karantzali et al., 2008; Orkin and Hochedlinger, 2011). Although the involvement of epigenetic mechanisms in self-renewal, pluripotency and regulation of differentiation has been extensively investigated (Atkinson and Armstrong, 2008), we are far from understanding phenomena such as the metastability of ESCs (Hayashi et al., 2008) and the heterogeneity of ESC-derived differentiated cells (Mummery et al., 1990). The concept that during embryo development the major epigenetic changes affecting genome regulation represents the conversion from a euchromatic to a heterochromatic compact state (Arney and Fisher, 2004) has been amply debated (Golob et al., 2008; Marks et al., 2012). As ESCs have the intrinsic ability to give rise to all differentiated tissues (Boiani and Schöler, 2005; Chambers and Smith, 2004; Golob et al., 2008; Marks et al., 2012; Solter, 2006), they represent a powerful model to decrypt both the mechanism(s) of pluripotency and the potential impact of epigenetic modulators on stem cell and differentiation potential (Arney and Fisher, 2004; Dovey et al., 2010; Hsieh et al., 2004; Karantzali et al., 2008; Kim et al., 2010; Lee et al., 2009; Lenka and Ramasamy, 2007). In this scenario, it is not unexpected that epidrugs act as modulators of stem cell potentiality and/or differentiation of ESCs. Indeed, the histone deacetylase inhibitor (HDACi) VPA (Valproic Acid) has been reported to impact on differentiation (Jergil et al., 2011; Lee et al., 2009) and the G9 methyl-transferase inhibitor, BIX01294, to affect reprogramming (Medvedev et al., 2011).

Here, we identify MS-275 (MS-275) a class I histone deacetylase inhibitor (HDAC) as a modulator of the differentiation potential of ESCs. We identified MS-275 in a cell-based screen as a regulator of mouse ESC proliferation and priming into differentiation. Notwithstanding, MS-275-induced ESC priming is fully reversible *in vitro*. MS-275-primed ESCs only give rise *in vivo* to benign teratomas, but not teratocarcinomas, with prevalence for neural-derived structures. Moreover, ESCs transiently treated with MS-275 were unable to colonize blastocysts. We thus suggest that the higher acetylation-state primes ESCs into neural-bound commitment.

## Results

### ESC proliferation is affected by chromatin modifying drugs

**Fig. 1.**
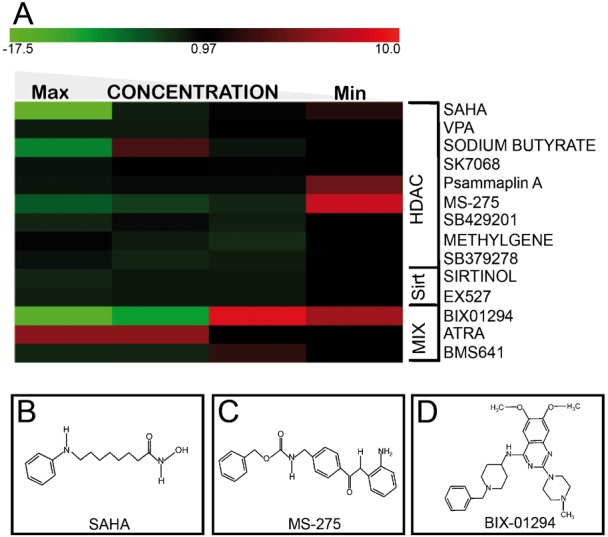
Effects of different drugs on ESC proliferation. (A) Mouse embryonic stem cells (TBV2) engineered for the expression of Enhanced Green Fluorescent Protein under the control of beta actin promoter (β-actin/EGFP TBV2) were plated in automation by using the Cellmaker and treated with the indicated drugs after 6 h. The fluorescence emitted was recorded after 42 h. The data were validated by semi-automated counts for MS-275, BIX01294 (supplementary material Fig. S1A). The columns are increasing concentrations of the compounds. The list of drugs and concentration is shown in supplementary material Table S1. (B–D) The structures of SAHA, MS-275, and BIX-01294, respectively.

To investigate the impact of epigenetic modulators on ESC growth, we screened a small selection of known epidrugs targeting diverse classes of chromatin enzymes using an integrated robotic workstation (Casalino et al., 2011). EGFP-marked mouse ESCs (β-actin EGFP-TBV2) were plated in feeder-free gelatin-coated 96-well plates and allowed to adhere for 6 hours before the addition of selected epidrugs at four different concentrations (supplementary material Table S1). Following 36 hours of culturing in the presence of the compound, EGFP-derived fluorescence was quantified as a proxy of cell proliferation. A subset of the results is represented as a heat map (Fig. 1A). All-Trans Retinoic Acid (ATRA), included as a positive control, showed the expected pro-proliferative effect as compared to the control (vehicle) (Fig. 1A). HDACis, such as Vorinostat (SAHA) (Butler et al., 2000) (Fig. 1B) and MS-275 (MS-275) (Park et al., 2004; Saito et al., 1999) (Fig. 1C), displayed a dose-dependent effect, being cytotoxic at higher doses and pro-proliferative at lower concentrations (supplementary material Table S1). A similar effect was obtained with BIX01294, a G9 methyltransferase inhibitor (HMTi) (Chang et al., 2009) (Fig. 1D). Validation by cell count confirmed these results (supplementary material Fig. S1A) and both SAHA and MS-275 displayed dose-dependent HDAC1 inhibition (supplementary material Fig. S1B).

**Fig. 2.**
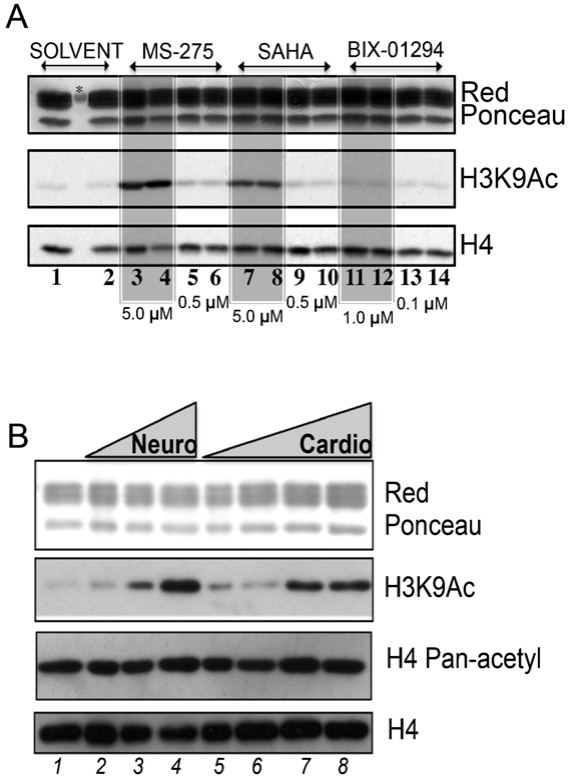
Histone acetylation upon chromatin modulator treatment and during ESC differentiation. (A) Western blot for H3K9 acetylation: lanes 1,2: DMSO; lanes 3,4: MS-275 at 5.0 µM; lanes 5,6: MS-275 0.5 µM; lanes 7,8: SAHA at 5.0 µM; lanes 9,10: SAHA at 0.5 µM; lanes 11,12: BIX-01294 at 1.0 µM; lanes 13,14: BIX-01294 at 0.1 µM. Odd and even numbers are at 12 h and 24 h, respectively. (B) Acetylation levels of H3K9 during neural and cardiac differentiation: lane 1) undifferentiated stem cell; lanes 2–4, neuronal differentiation at 4, 8 and 10 days, respectively. Lanes 5–8: at 4, 8 and 10 and 13 days. The H4pan antibody recognizes K 4-7-11-15ac. Histone H4 and Ponceau Red are used as loading controls. Asterisk represents the molecular weight marker.

Treatment of ESCs (or β-actin EGFP-TBV2 cells) with SAHA or MS-275 for 12 h and 24 h strongly increased acetylation of H3K9 (Fig. 2A), H3K18 and H3K23 (supplementary material Fig. S2A,B). Interestingly, a physiological increase of H3K9 acetylation, i.e. in absence of any ‘epidrug’ treatment, was also observed during neural and cardiac differentiation (Fig. 2B), suggesting that increased acetylation might impact on ESC differentiation potential.

### Transient MS-275 treatment promotes neural differentiation of ESCs *in vitro*

**Fig. 3.**
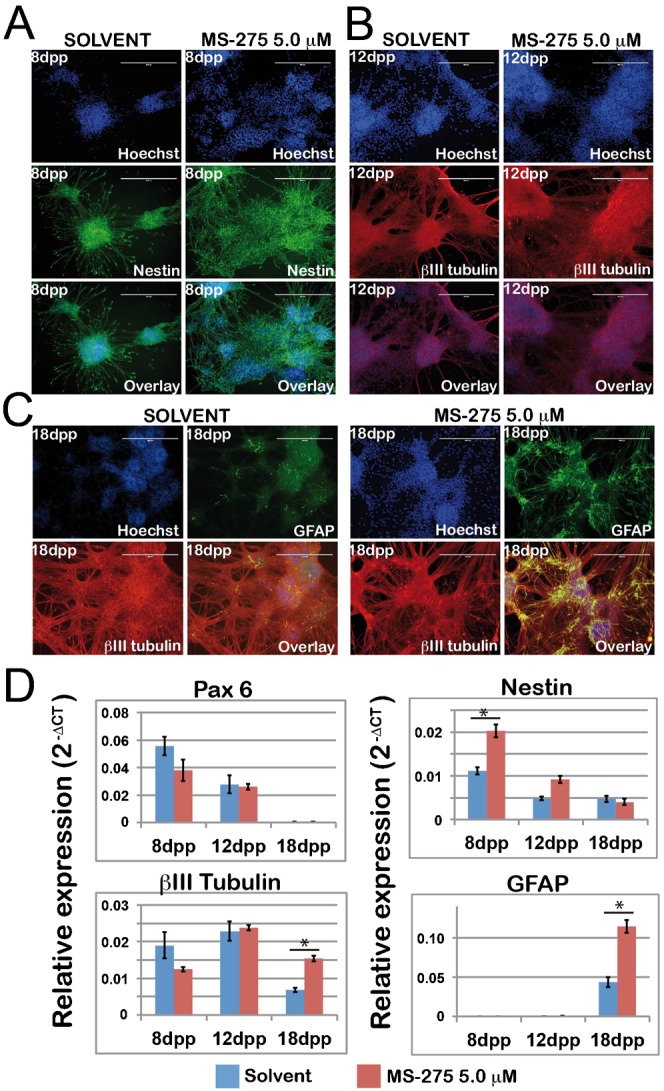
MS-275 impacts on neural differentiation of ESCs *in vitro*. ESCs were pretreated for 24 hours with solvent (DMSO 0.1%) or 5 µM MS-275 and then re-plated for neural differentiation. (A–C) Immunostaining at 8, 12 and 18 day post plating (dpp) of ESC neuronal differentiation, respectively. Cells were fixed and stained with antibodies specific for Nestin, βIII-tubulin and GFAP, showing the presence of neural progenitors, mature neurons and glial cells, respectively. Nuclei were counterstained with Hoechst. Phase contrast (PC) and overlay fluorescence picture are shown. Scale bars: 400 µm. (D) RT-qPCR analysis of neural markers: neuro-ectoderm (Pax6) neural progenitors (Nestin), mature neurons (βIII-tubulin) and Glia (GFAP). mRNA was normalized to GAPDH. Data are means of three independent experiments (error bars: ± S.D.). *p<0.001.

To evaluate the ability of acetylation to modulate ESC differentiation *in vitro*, TBV2 cells were pre-treated for 24 hours with MS-275 (5.0 µM) or DMSO (0.1%) as control vehicle, and then induced to neuronal differentiation, as previously described (Fico et al., 2008) (Fig. 3A–C). At day 8 of differentiation, Nestin-positive rosette-like clusters, typical of neural precursor, were much more expanded in ESC MS-275-pulsed -derived progeny compared to control. Although the beta-III-tubulin-positive mature neurons with complex neurite branching or outgrowth were clearly detectable at day 12 and 18 of differentiation for both solvent and MS-275-pulsed cells, MS-275-pulsed cells yielded more GFAP-positive glial cells. RT-qPCR data confirmed the difference observed in the relative abundance of neural progenitors and glial cells. The MS-275-pulse caused a slight decrease of the neuroectodermal marker *Pax6* expression, accompanied by an earlier, and more sustained expression of *Nestin* (Fig. 3D). Small differences until the day 12 of differentiation in βIII-tubulin levels were observed; in contrast at day 18, a higher level after the treatment is detectable. In addition, the RT-qPCR data confirm and strengthen the strong increase of GFAP in treated cells, already observed with immunohistochemistry (Fig. 3C,D).

### MS-275 treatment reversibly impacts on stem cell potentiality *in vitro*

**Fig. 4.**
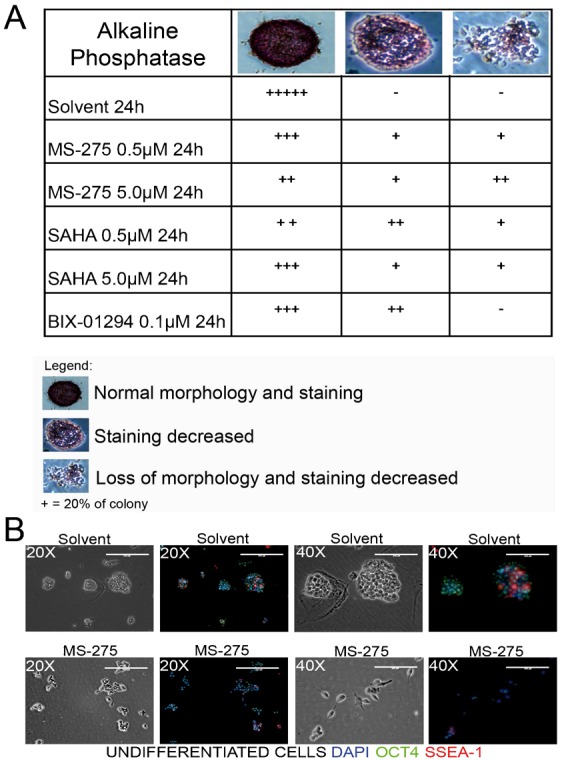
HDAC inhibitors modulate ESC stem cell potential at morphological and molecular level. (A) Effect induced by the indicated compounds on ESC colony morphology and alkaline phosphatase staining. (B) Immunofluorescence of ESC stained for DAPI-OCT4-SSEA-1. Upper panel: control; lower panel: MS-275 at 5.0 µM for 24 h.

**Fig. 5.**
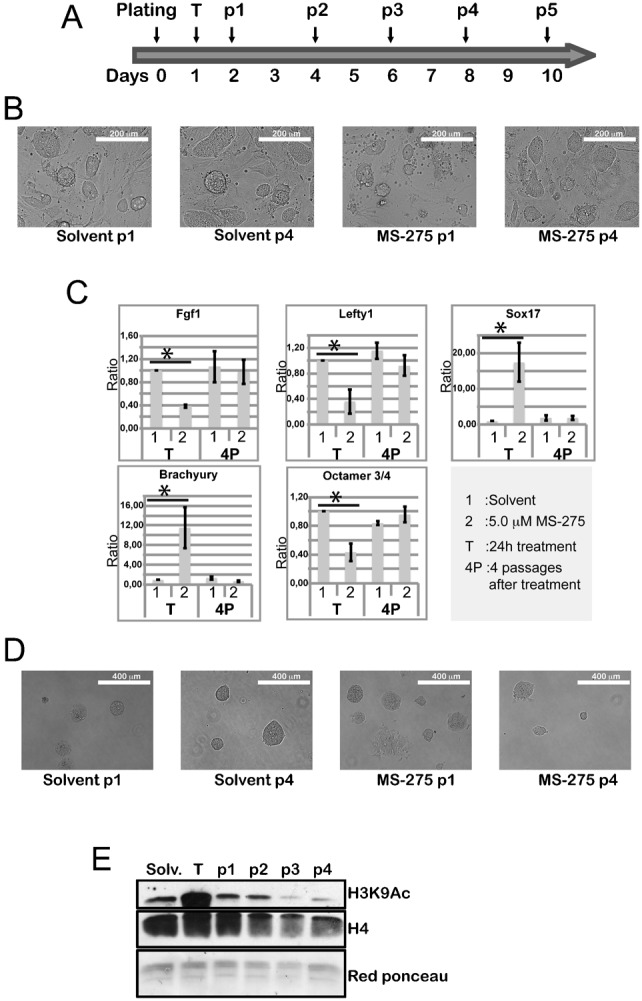
Reversible effect of MS-275 on ESCs. (A) Schematic representation of the experiment: T  =  treatment of 24 h with 5.0 µM MS-275; p1–p4: days of medium change and photo collection. (B) Photo in bright field of solvent and drug treatment at p1, p4. (C) q-PCR of solvent and MS-275 at 5.0 µM concentration treated cells at p1 and p4 for the indicated markers; *p<0.01. (D) Colony formation assay for each day and treatment. (E) H3K9 Acetylation (Ac) levels after 24 h of treatment with MS-275 at 5.0 µM and at the different passages (p1, p2, p3 and p4). Scale bars: 200 µm (B), 400 µm (D).

The fact that the H3K9ac level induced by the HDACis MS-275 or SAHA is reminiscent of the strong increase of H3K9ac observed during ESC differentiation (Fig. 2B) and that MS-275 treatment impacts on neural differentiation (Fig. 3A–C) prompted us to investigate whether the epidrug-treated ESCs still display a pluripotent status using the alkaline-phosphatase (AP) colony formation assays (O'Connor et al., 2008) (Fig. 4A). With this aim, TBV2 cells pretreated for 24 hours with the three selected epidrugs (SAHA, MS-275, BIX01294) were plated at low density and the colony morphology was observed at day 4 after plating. Based on both AP staining and colony morphology, the colonies were classified into 3 main categories: i) undifferentiated colonies with well defined, rounded shape and strong AP staining; ii) colonies with low AP staining retaining defined edges and rounded features and iii) AP negative colonies showing a flat morphology. While, as expected, almost 100% of the control colonies displayed a characteristic round-shape morphology, nearly 40% of the colonies showed a flat phenotype with irregular edges and loss of AP staining after treatment with 5.0 µM MS-275 (third lane, Fig. 4A). Immunofluorescence analysis showed reduced expression of the pluripotency markers OCT4 and SSEA-1 in ESCs treated with MS-275 5.0 µM compared to the control (Fig. 4B), thus corroborating the potential decrease of stem cell potential despite the presence of LIF in the culture medium. Taken together, these findings showed that pre-treatment with MS-275 causes loss of pluripotency features and primed ESCs for differentiation. Interestingly, when MS-275 was transiently applied for 24 hrs and then washed out and cells re-plated and propagated for 4 passages (p1–p4) (Fig. 5A) with medium change every two days, by passage 4 MS-275-treated cultures regained the morphological (clonogenicity and colony shape) and molecular (expression of the ESC-associated genes *Oct3/4* and *Lefty1* and downregulation of differentiation markers *SOX17* and *Brachyury*) features of ESCs (Fig. 5A–D), thus suggesting that MS-275-induced biological effect was fully reversible. In full agreement, modulation occurring at H3K9Ac post MS-275 treatment (Fig. 5E) and ESC proliferation were also reversible. Similar results were obtained using MS-275 at 0.5 µM (supplementary material Fig. S3). All together our findings support the hypothesis that MS-275-induced hyperacetylation impacts on ESC pluripotency potential and point to a fully reversible effect of this epidrug.

### Expression analysis of MS-275-primed ESCs reveals commitment to neural differentiation

**Fig. 6.**
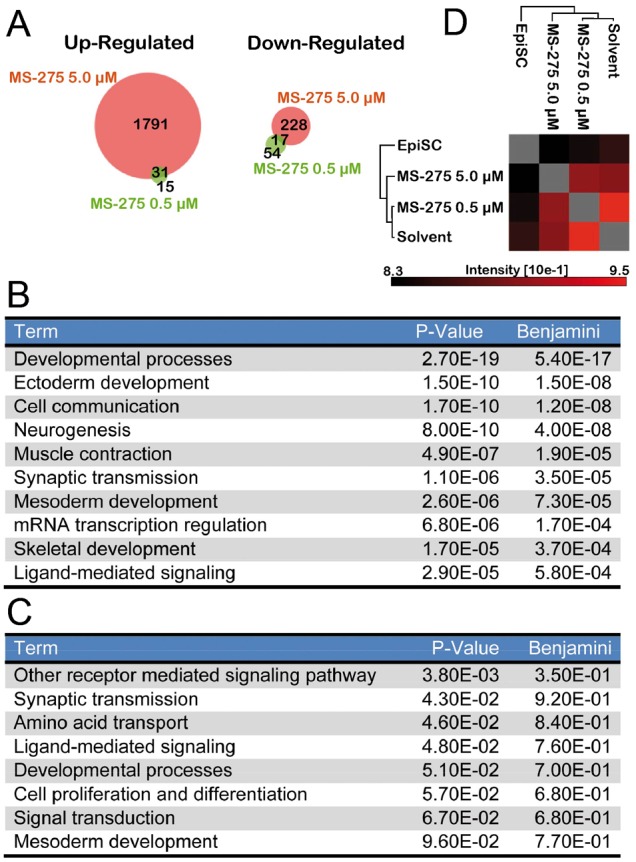
Gene expression profiles suggest neural orientation upon MS-275 treatment. (A) Venn diagram of up- and downregulated genes of 0.5 µM and 5.0 µM MS-275 versus solvent, respectively. (B,C) Gene ontology results for up- and downregulated genes, respectively. (D) Spearman Rank Correlation between MS-275 5.0 µM treated TBV2 and Epi-SCs.

Transient MS-275 modulation of stem cell and differentiation markers (Fig. 5) prompted us to apply a Differential in Gel Electrophoresis (DiGE) followed by “In Situ Digestion” and nano LC-MS/MS interpretation (Mascot search engine) to determine protein targets. Almost 200 proteins were identified with differential expression after 5.0 µM MS-275 (only mild fluctuations with the lower MS-275 concentration, supplementary material Figs S4, S5). GO analyses indicated modulation of differentiation and neural-oriented pathways (supplementary material Fig. S5; Table S2), yet, the previously identified markers (Fig. 5A–D), were not among them. To further investigate differential expression, two replicas of genome-wide transcriptome profiles (RNA-seq) were generated from cells treated for 24 hours with vehicle or MS-275 at 5 µM in presence of LIF. The data (GEO GSE45909) were analysed using the Genomatix tool and normalized expression values were compared between vehicle and treated at 0.5 µM and 5.0 µM. 1822 genes had higher expression values in the MS-275 treatment at 5.0 µM, whereas only 245 genes were downregulated. The treatment with 0.5 µM MS-275 yielded to 46 up- and 66 downregulated. Venn diagram shows the overlap of up- and downregulated genes between the two conditions (Fig. 6A). Moreover, David tool (http://david.abcc.ncifcrf.gov) was used for GO enriched terms; results are shown (Fig. 6B,C). Within the most enriched terms were ectoderm and neurogenesis and included neural lineage precursor genes such as *Nestin, Hey1* and *2*, *Foxn4*, *Id2* and *4*. Clearly, 5.0 µM MS-275 induced loss of pluripotency markers (Fig. 5) and enhanced expression of differentiation-related genes, in particular in ectoderm and neural differentiation (Fig. 6). Interestingly, *Fgf1, Lefty1 SOX17* and *Brachyury* were modulated as previously found (Fig. 5), thus corroborating and extending the evidences that MS-275 primes ESC versus neural differentiation. The small number of up- and downregulated genes in the treatment condition of 0.5 µM did not allow a statistically sound interpretation. Given that Epiblast Stem Cells (EpiSCs) have been described as ESC with a specific transcriptome and incapacity to colonize blastocysts (Bernemann et al., 2011), we also compared the expression profile of TBV2 cells (with and without MS-275) to the Epi-SC profile using MultiExperiment Viewer (http://www.tm4.org/mev.html) (Fig. 6D). The pairwise comparison of non negative matrix factorization based Spearman rank correlation demonstrated that MS-275 treated ES cells are more closely related to untreated ESC that to EpiSC.

### *Transient* MS-275-treatment impacts on ESC-derived teratoma formation and composition *in vivo*

**Fig. 7.**
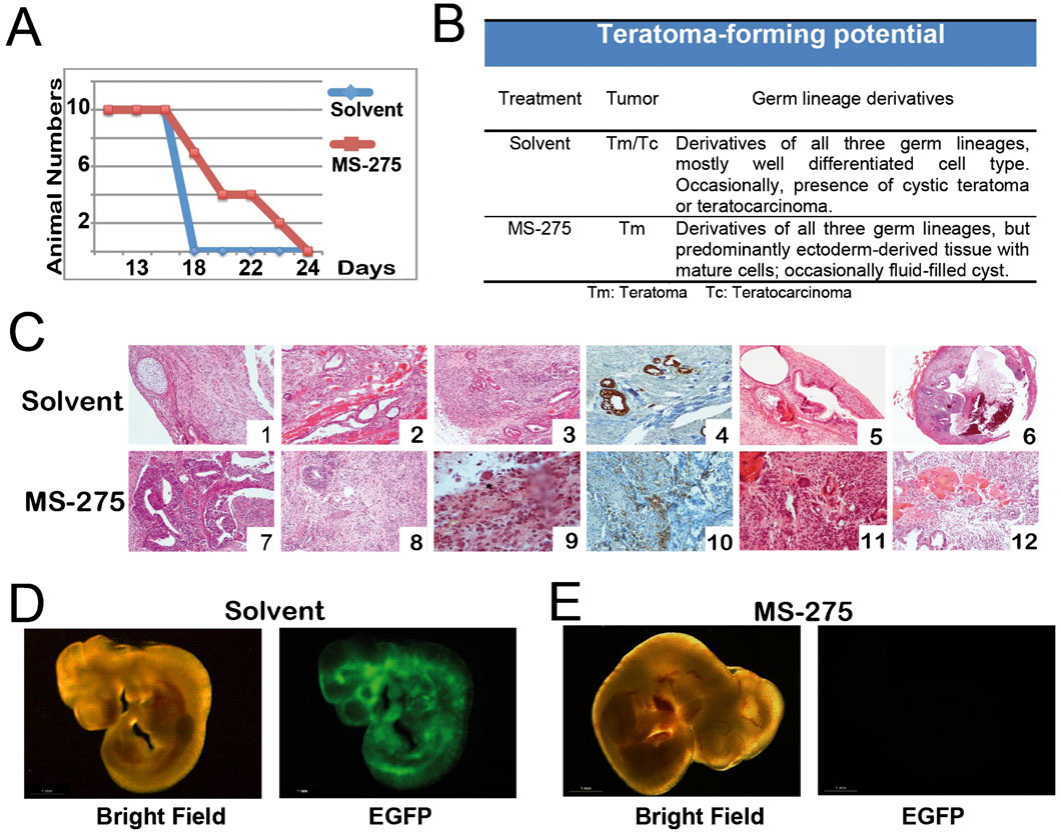
MS-275 *in vitro* priming impacts both on terato-carcinoma development and content as well as blastocysts colonization *in vivo*. (A) Teratoma growth rate; *x*-axis: days of life of each mouse; *y*-axis: number of mice alive. (B) Post-surgery analysis of teratoma composition. (C) Anatomic/pathologic evaluation. Upper panels: *control* (ctr); lower panels: lesions obtained in teratomas derived from *in vitro* 5.0 µM MS-275 pre-treated cells. 1: cartilage and respiratory epithelium; 2: respiratory epithelium and smooth muscle; 3: neuron tubes; 4: immunohistochemistry of pan-cytokeratin, demonstrating the epithelial origin of the structure; 5,6: teratocarcinoma tumor histo-pathological pattern; 7,8: neuron tubes; 9: a mature neuron (see asterisk); 10: immunohistochemistry for GFAP showing glia formation; 11,12: teratoma tumor histopathological pattern. (D,E) Chimera formation assay. Representative photomicrographs (fluorescence/bright field) of the embryos (E11) obtained after blastocyst injection with engineered TBV cell treated with MS-275 (5.0 µM) (E) and solvent (D).

When introduced into immunodeficient mice, ESCs spontaneously form teratoma-like masses, containing ectoderm-derived (such as nerve and skin), mesoderm-derived (bone, blood, and muscle) and endoderm-derived (gut) tissues (Thomson et al., 1998). As the formation of differentiated tissues from all the three somatic germ layers in the teratomas is taken as one of the pluripotency indicators characteristic for ESCs (Lensch et al., 2007), we tested the teratoma-forming ability of the MS-275-treated ESCs. ESCs were exposed to MS-275 (5.0 µM) for 24 h, or to vehicle as control, and injected subcutaneously into the flanks of SCID mice. Given the different growth kinetics, animals were sacrificed at different times (Fig. 7A) depending on the tumor dimension. Due to reduced tumor dimension, some animals were sacrificed after 22 days from cells injection. The histopathological analysis showed that control tumours displayed different degrees of differentiation and tissues types (Fig. 7C1–4), as well as three-dimensional teratocarcinoma formation (Fig. 7C3–6). Importantly, *in vitro* MS-275 pretreated cells failed to form teratocarcinomas developing only well-differentiated teratomas, with a prevalence of epithelial and neural tissues, such as small neuronal tubes (Fig. 7C8,9) and absence of respiratory epithelium, cartilage and smooth muscle, compared to control. Histo-pathological conclusions are summarized in Fig. 7B. Taken together these data suggest that MS-275-pretreated ESCs, despite retaining the ability of teratomas formation with a preponderance of neural lineage-specific tissues (neuroectodermal structures), had lost the potential of forming malignant teratocarcinomas.

### Dose-dependent effect of *in vitro* MS-275 treatment on ESC contribution to embryo development

Given that pre-treatment with MS-275 reduced the pluripotency potential of ESCs *in vitro*, which maintain the ability to generate teratomas *in vivo*, we questioned whether their contribution to embryo formation might be compromised. To this end, EGFP-TBV cells were pretreated *in vitro* with 0.5 and 5.0 µM MS-275 for 24 hours, or with vehicle as control. After washout of solvent and MS-275, cells were injected in about 30 blastocysts and transferred to the uterus of pseudo-pregnant female recipient CD-1 mice. Interruption of mice pregnancy at day 11 (E11) post-coïtum (d.p.c.) was performed, embryos were dissected and images captured using a fluorescence stereomicroscope. While cells pretreated *in vitro* with solvent and 0.5 µM MS-275 (data not shown) still contributed to the entire embryo, *in vitro* pre-treatment with 5.0 µM MS-275 showed absence of EGFP signal in the embryo (Fig. 7D,E; supplementary material Table S3). This observation corroborates and extends our conclusion that *in vitro* transient exposure to 5.0 µM MS-275 affected pluripotency and/or ESC differentiation potential *in vivo*, thus affecting final tissue formation.

## Discussion

The effects of chromatin modulators (epidrugs) on stem cell potentiality and differentiation have been poorly addressed. This topic is, however, of particular relevance given that epidrugs are entering the clinics and their potential action on adult stem cells might influence both treatments and time of patient's recovery. Moreover, a bottleneck of the use of embryonic stem cells in therapy is the risk of teratocarcinoma formation, thus suggesting that tools aiming at prevention of formation of ESC-derived tumors might increase their therapeutic applications.

The notion that the differentiation potential of pluripotent embryonic stem cells (ESCs) is highly affected by their epigenetic status together with the availability of several epi-enzyme modulators (epidrugs) led us to hypothesize that epidrugs might be useful tools to modulate ESC differentiation. Supporting this hypothesis, recent studies suggest that Vorinostat, BIX-01294 and Tricostatin A (TSA) may play a role in the ESC differentiation (Milde et al., 2011; Shi et al., 2008). Here we show that the acetylation levels of H3K9ac (Fig. 2A) as well as for H3K23ac and H3K18ac (Wang et al., 2008), specifically increase during key steps of ESC differentiation and that treatment of ESCs with HDACi such as MS-275, mirrored this phenomena. We further showed that MS-275 initially caused an increase followed by a decrease of proliferation reminiscent of maturation processes. That in this setting MS-275 behaved similarly to SAHA (Vorinostat) is a point of particular interest given that i) Vorinostat has been reported to alter ESC pluripotency (Park et al., 2011) and ii) only class I HDACs (and mainly HDAC1 and 2) are inhibited by MS-275, thus restricting the epi-targets potentially responsible for this effect. Moreover, the concentration dependent dual growth effect – positive and negative proliferative action displayed by some epidrugs amongst which MS-275 – correlates the growth modulation with the enzyme inhibition suggesting target specificity. Thus, the HDACi treatment seems to recapitulate the hyper-acetylation state characteristic of specific steps of differentiation (Fig. 2B) strongly suggesting that HDACi cause loss of stem cell potential and modulate induction of differentiation.

Whether the hyper-acetylated state of chromatin occurring during neural and cardiac differentiation (Fig. 2B) represents a causal step or an effect of induction of differentiation represents a crucial point of reflection. Interestingly, in mouse ESCs, a hyper-acetylated state is reached both in neural and cardiac differentiation with different time courses, being clearly visible at day 8 and 10 in neural and cardiac differentiation, respectively. Despite the different settings, these observations might be interpreted in diverse ways. The timing itself may be crucial; neural differentiation might simply need increased acetylation at an earlier time point compared to cardiac differentiation. Alternatively or in addition, hyper-acetylation might be induced in distinct chromatin areas in the two differentiation programs, which eventually induce hyper-acetylation of different chromatin areas. Obviously, both options might co-occur, being both time of acetylation and areas of acetylation key events to be executed. In the same time result very interesting the capability of ESCs to revert phenotype and biochemistry effect due to MS-275 treatment.

In agreement with the hypothesis that HDACi targeting HDAC1 and 2 might influence differentiation, a transient, one-day pulse of MS-275 pre-treatment (to a higher extent than SAHA) altered the colony morphology of ESCs even in the presence of LIF supporting an active role of MS-275 for differentiation commitment and highlighting the option that timing of hyper-acetylation might play a role in pluripotent stem cell differentiation. In accordance, the treatment of ESCs with MS-275 (in presence of LIF) primed differentiation predominantly along neural lineages. Indeed, RNA-Seq analysis revealed a significant neural-oriented modulation together with a decrease of embryonic functional genes, suggesting an active modulation of HDACi vs ESC differentiation as well as corroborating that temporal frame of hyper-acetylation might actively prime a neural-oriented fate. Interestingly, teratoma formation *in vivo* indicates that pluripotency is largely unaffected in MS-275-primed ESCs given that teratomas are present in all animal groups, although HDACi priming caused a preponderance of differentiated tissues towards a neuronal phenotype. Whether the preponderance towards neuronal differentiation *in vivo* is due to induction of neural differentiation as a consequence of the MS-275 treatment and/or to the repression of other differentiation programs remains to be established. Importantly, MS-275 treatment and likely the acetylation status of ESCs at the moment of the injection altered the cancer potential within teratomas given that no malignant teratomas were formed. Most remarkably, MS-275-treated ESCs were unable to contribute to hybrid embryos similar to Epiblast Stem Cells (EpiSC) (Bernemann et al., 2011). However, the expression profiles of treated ESCs are more similar to untreated ESCs than to EpiSC.

In conclusion, given the potential of pluripotent stem cells in biomedical applications, a better comprehension of the mechanism(s) of differentiation both *in vitro* and *in vivo* and insights into potential pharmacological approaches to modulate differentiation may represent the road ahead for personalized medicine.

## Materials and Methods

### Cell culture

The feeder-dependent mouse embryonic stem cells TBV2 (129/SvP; wild type or EGFP-transgenic) have been used throughout the study and maintained in culture as described previously (Casalino et al., 2011).

### Compounds

A collection of compounds was chosen for screening analysis (Álvarez et al., 2009; Bieliauskas and Pflum, 2008; Göttlicher et al., 2001; Grozinger et al., 2001; Hu et al., 2003; Kubicek et al., 2007; Lin et al., 1998; Park et al., 2008; Richon et al., 1996; Sealy and Chalkley, 1978; Zhang et al., 2009). The majority of compounds were purchased from Sigma–Aldrich; psammaplin A and UVI5008 were obtained by Prof. A. de Lera, BMS641 was a kind gift of BMS Pharma, SAHA was a kind gift of Merck and MS-275 was purchased from Alexis. Diamide was included as negative control. Details are described in supplementary material Table S1. For mother plate preparation, compounds were diluted in PBS 1×. Compounds were added randomly at 96 multi-wells plates in tripled, excluding external side of plate, which is filled with PBS 1×.

### ESC proliferation assay and counting

ESC-EGFP were counted and suspended at 10^5^ cells/mL, from which 10^4^ cells/well were seeded in 96 gelatin multi-well plates, for a total volume of 100 µL. Cells were left adhere for 12 hours and then compounds were added. After 36 hours from treatment the cell layer was washed and fluorescence measured with TECAN Infinite M200.

For cell counting, TBV2 cells were seeded in duplicate at a density of 250,000 cells/well in 6 multi-well plates (this is equivalent at adopted density for the screening in MW96). Medium culture was changed after 24 hours, and, at stimulation of 36 hours, cells were removed, diluted in Trypan blue (Sigma) and counted.

### HDAC assay

The assay was performed as described previously (Nebbioso et al., 2011).

### Protein extraction, Western blots

Cells were lysed in 200 µL of lysis buffer (Tris–HCl 50 mM, NaCl 150 mM, NaF 10 mM, NP-40 1.0%) with 10 µL/mL of PIC (Sigma–Aldrich) and 200 µM of PMSF (Sigma–Aldrich). Proteins were quantified using “Bio-rad protein assay”. Histone extraction and Western blots were performed as described previously (Nebbioso et al., 2009). Antibodies used: H3 pan-Acetyl (UPSTATE 06-599); H4 pan-Acetyl (UPSTATE 06-866); H3K9Ac (AB4441); H3 (AB1791); H4 (AB7311); H3K12ac (AB1191); H3K4me (AB8895); H3K9me (AB9045); HDAC4 (AB1437); HDAC5 (AB47519); HDAC2 (AB7029); HDAC4-6 (SIGMA H2287); HDAC1 (SC7872); HDAC3 (AB70030); H3K9me (AB9045); H3K4me2 (AB32356); H3K4me3 (AB8580); H3K9me2 (AB1220); H3K9me3 (AB8898); H3R17me2 (AB32356).

### Colony formation assay

Cells were plated at low density (1000 cell/cm^2^) on gelatin plates in a medium for undifferentiated ESCs. Compounds were used as indicated. After 5 days cells were fixed (4% paraformaldehyde in PBS 1×) for 2 minutes. Then, cells were washed in rinse buffer 1× (20 mM TRIS–HCl, pH 7.4, 0.15 M NaCl, 0.05% Tween-20). Alkaline Phosphatase Detection Kit (Chemicon International) was used for staining. Cells counted at microscopy as colonies number expressing alkaline phosphatase.

### RNA isolation, reverse transcription, RNAseq and qPCR analysis

The experiments were performed as described previously (Marks et al., 2012). Briefly total RNA was isolated with Trizol (Invitrogen) and 100 µg total RNA was subjected to two rounds of poly(A) selection (Oligotex mRNA Mini Kit; QIAGEN), followed by DNaseI treatment (QIAGEN). 100–200 ng mRNA was fragmented by hydrolysis and purified (RNAeasy Minelute Kit; QIAGEN). cDNA was synthesized with 5 µg random hexamers by Superscript III Reverse Transcriptase (Invitrogen). Ds cDNA synthesis was performed according to the manufacturer's recommendations and purified (Minelute Reaction Cleanup Kit; QIAGEN).

Sample preparation, cluster generation and sequencing (36 bp) was performed with the Illumina Genome Analyzer IIx (GAIIx) platform according to standard Illumina protocols. All sequencing results were mapped on the Mus musculus NCBI m37 genome assembly (MM9; assembly July 2007). Normalized expression values were obtained by Genomatix (http://www.genomatix.de). Functional annotation analyses were performed using the Panther DB tool for biological pathway (http://www.pantherdb.org), GO enrichment analysis was obtained using DAVID (http://david.abcc.ncifcrf.gov). Raw data have been deposited on GEO: GSE45909.

### Protein analysis on 2D gels

Protein total extracts from TBV2 treated for 24 h with 0.5 µM MS-275, 5.0 µM MS-275 or solvent were prepared. The mixture containing an equal aliquot of all samples has been labelled with the fluorescent dyes Cy2, Cy3 and Cy5. Experiments were performed as described previously (Cimmino et al., 2007).

### Mass spectrometry and protein identification

Matched spots of interest were picked manually from the preparative gel. These spots were subjected to in-gel trypsinization according to the manufacturer's protocol (Promega, USA). After overnight digestion, buffer containing the peptides was recovered. Additional extraction of peptides was carried out with 100 µl of 50% acetonitrile in 1% formic acid. The extracts were poled and vacuum-dried. For LC-MS/MS, peptide mixtures were dissolved in 50% acetonitrile and 1% formic acid solution and analyzed with nano-LC system (Applied Biosystems). Some spots were analyzed by off-line nano-spray method. These peptides were dissolved in 20 µl of 50% acetonitrile in 0.1% formic acid. Nano-spray ionization was carried out using an ion spray voltage of 900. The spectra were acquired in an information dependent manner with Analyst QS 2.0 software to generate raw data. Database searching was completed using Mascot search program (Version 1.6, Matrix Science, UK). Search parameters were as follows: 1 missed cleavage allowed, carbamidomethylation set as fixed modification, methionine oxidation as variable modification, peptide mass tolerance ±1.2 Da, fragment mass tolerance ±0.6 Da, monoisotopic mass values. Spectra were searched against NCBI or MSDB database. Criteria for positive identification were a significant Mascot probability score (score >40; p<0.05). Validated protein identification results are handed out in form of an Excel file. Analysis of modulated proteins was performed with String software (http://www.string-db.org).

### Teratoma formation

For teratoma formation, 7–8-week-old Fox Chase SCID mice (Charles River, Chatillon-sur-Chalaronne, France) were used. MS-275- or vehicle-treated ESCs were resuspended in PBS (without Ca^2+^ and Mg^2+^) to a concentration of 1.5×10^7^ cells/ml. 3×10^6^ cells were injected subcutaneously in the flank of ten animals per group. Tumor growth was monitored by three-weekly measurements of tumor diameters with a caliper. Tumor volume (TV) was calculated according to the following formula: TV (mm^3^) = d^2^*D/2, where d and D are the shortest and the longest diameters, respectively. All the animals injected developed the tumor. Mice were sacrificed when tumors reached the average volume of one cm^3^ in controls, and 0.35 cm^3^, when MS-275 pretreated cells were used. Tumors were paraformaldehyde-fixed and embedded in paraffin following standard procedures. Five micrometer-thick deparaffined tumor sections were stained with hematoxylin–eosin for the histological analysis. For immunohistochemistry, all samples were processed with the standard streptavidin–biotin-immunoperoxidase method (DAKO Universal Kit, DAKO Corp., Carpinteria, CA, USA). Monoclonal antibody for Glial Fibrillary Acidic Protein (GFAP) (DAKO; Clone 6F2) and polyclonal antibody Rabbit Anti-Cytokeratin (DAKO) were used at 1:100 dilution for 1 hr. Diaminobenzidine was used as the final chromogen, and hematoxylin as the nuclear counter stain. Negative control experiments for each tissue section were performed in the absence of the primary antibody. Positive controls were included in each experiment. The care and husbandry of mice and xenograft tumor experimental procedures were in accordance with European Directives no. 86/609, and with Italian D.L. 116. All the experiments have been approved by the Institute of Genetics and Biophysics veterinarian.

### Blastocyst injection

About 30 ESCs (vehicle or MS-275 *in vitro* pre-treated)/blastocyst were microinjected, following standard procedures.

## Supplementary Material

Supplementary Material
