# Supplementary material for: The class I-specific HDAC inhibitor MS-275 modulates the differentiation potential of mouse embryonic stem cells
Source: Biol Open. 2013 Aug 22;2(10):1070–7. doi: 10.1242/bio.20135587 (PMC3798190; doi:10.1242/bio.20135587)
Supplement: Supplementary Material [file supp_2_10_1070__index.html]

The class I-specific HDAC inhibitor MS-275 modulates the differentiation potential of mouse embryonic stem cells — The class I-specific HDAC inhibitor MS-275 modulates the differentiation potential of mouse embryonic stem cells — Supplementary Material 

# The class I-specific HDAC inhibitor MS-275 modulates the differentiation potential of mouse embryonic stem cells

## bio.20135587 Supplementary Material

**Files in this Data Supplement:**

- Supplementary Material - Gianluigi Franci et al. doi: 10.1242/bio.20135587
