# Supplementary material for: The class I-specific HDAC inhibitor MS-275 modulates the differentiation potential of mouse embryonic stem cells
Source: Biol Open. 2013 Aug 22;2(10):1070–7. doi: 10.1242/bio.20135587 (PMC3798190; doi:10.1242/bio.20135587)
Supplement: Supplementary Material [file supp_bio.20135587_bio.20135587-s1.pdf]

Supplementary Material

Gianluigi Franci et al. doi: 10.1242/bio.20135587

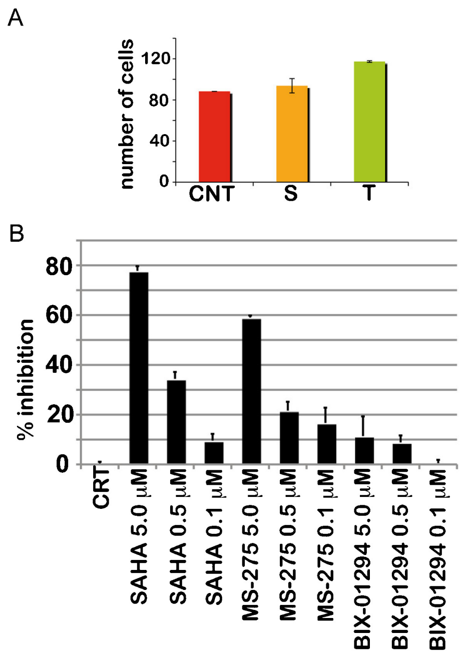

Fig. S1. Effect of MS-275 on proliferation of ESC and validation panels for HDAC activity *in vitro* and in cells. (A) Manual count of ESC treated with MS-275 for 24 h compared with solvent and untreated cells. (B) *In vitro* assay for HDAC1 inhibition upon the indicated treatments.

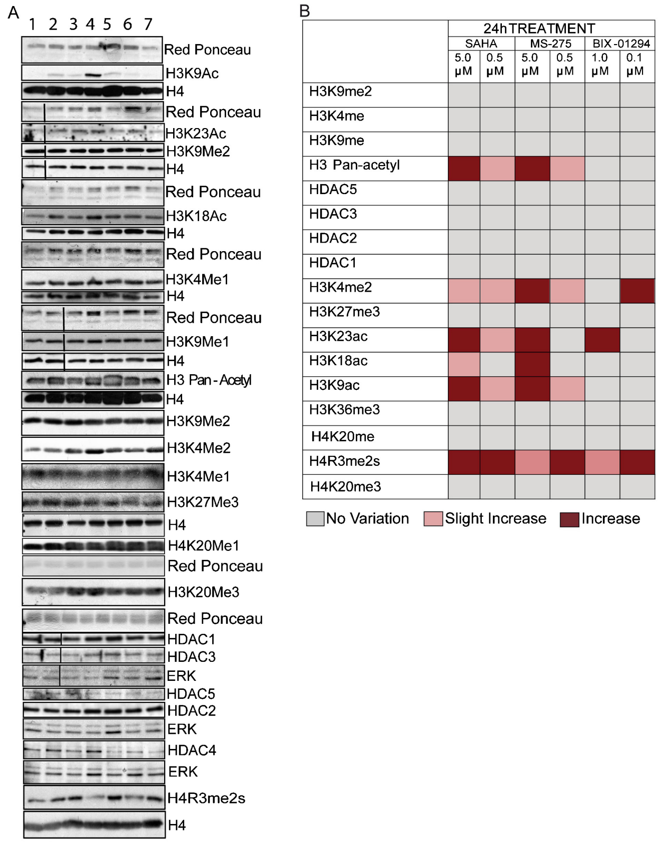

Fig. S2. Effect of epidrugs on histone modifications and HDACs expression in ESC. (A) Western blot of the indicated targets upon the following treatments: 1 (control), 2 (5.0 µM SAHA 24 h), 3 (0.5 µM SAHA 24 h), 4 (5.0 µM MS-275 24 h), 5 (0.5 µM MS-275 24 h), 6 (1.0 µM BIX-01294 24 h), 7 (0.1 µM BIX-01294 24 h); (B) heat map of semi-quantitation of histone marks and HDACs upon the indicated treatments.

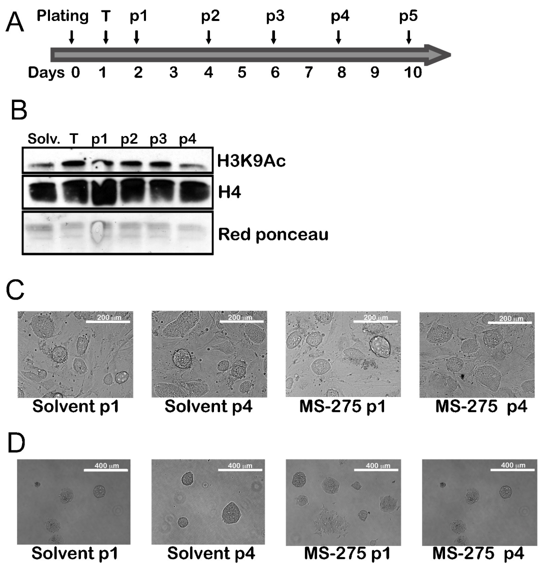

Fig. S3. Stem cell potential impact of 0.5 µM MS-275 treatment on ESC. (A) Experiment scheme; T: treatment 24 h 0.5 µM MS-275; p1–p4 days of medium change and cell photo collection. (B) Western blot for H3K9 acetylation (Ac) after 24 h of MS-275 treatment at concentration of 0.5 µM and acetylation levels at different passages (p1, p2, p3 and p4) after the same treatment. (C) Photo in bright field of solvent and drug treatment at p1 and p4. (D) Colonies formation assay for each day and treatment. Scale bars: 200 µm (C), 400 µm (D).

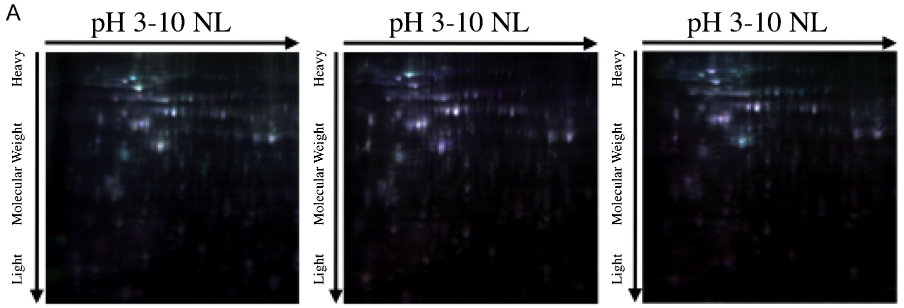

Gel 1 (Master)	Solvent-Cy3	MS 5.0 µM 24h-Cy5	Mix-Cy2
Gel 2	Solvent-Cy3	MS 0.5 µM 24h-Cy5	Mix-Cy2
Gel 3	MS 5.0 µM 24h-Cy3	Solvent-Cy5	Mix-Cy2

	Down-regulated	Up-regulated	TOT
MS-275 0.5µM 24h Vs Solvent	14	27	41
MS-275 5.0µM 24h Vs Solvent	89	107	196

Down-regulated: Ratio > - 1.20 p-value ≤ 0.1

Up-regulated: Ratio > 1.20 p-value ≤ 0.1

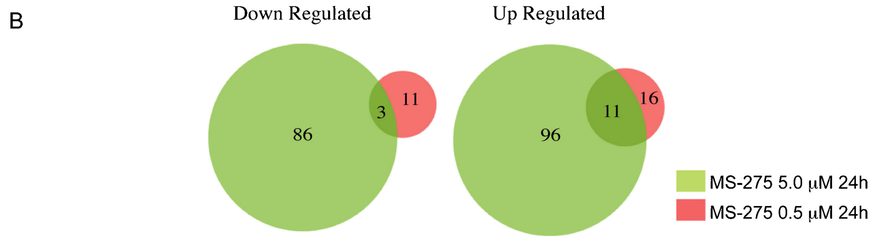

Fig. S4. Difference in gel electrophoresis analysis of MS-275 effect on ESC. (A) 2D-PAGE gels of total protein extracts from ESC treated with MS-275 at 0.5 and 5.0 µM. For sample labeling see figure scheme. (B) Venn diagrams displaying numbers of up- and downregulated proteins (p-value of 0.009); 5.0 µM MS-275: downregulated 89 (86+3), upregulated 107 (96+11) proteins; 0.5 µM MS-275: downregulated 14 (11+3) and upregulated 27 (11+16).

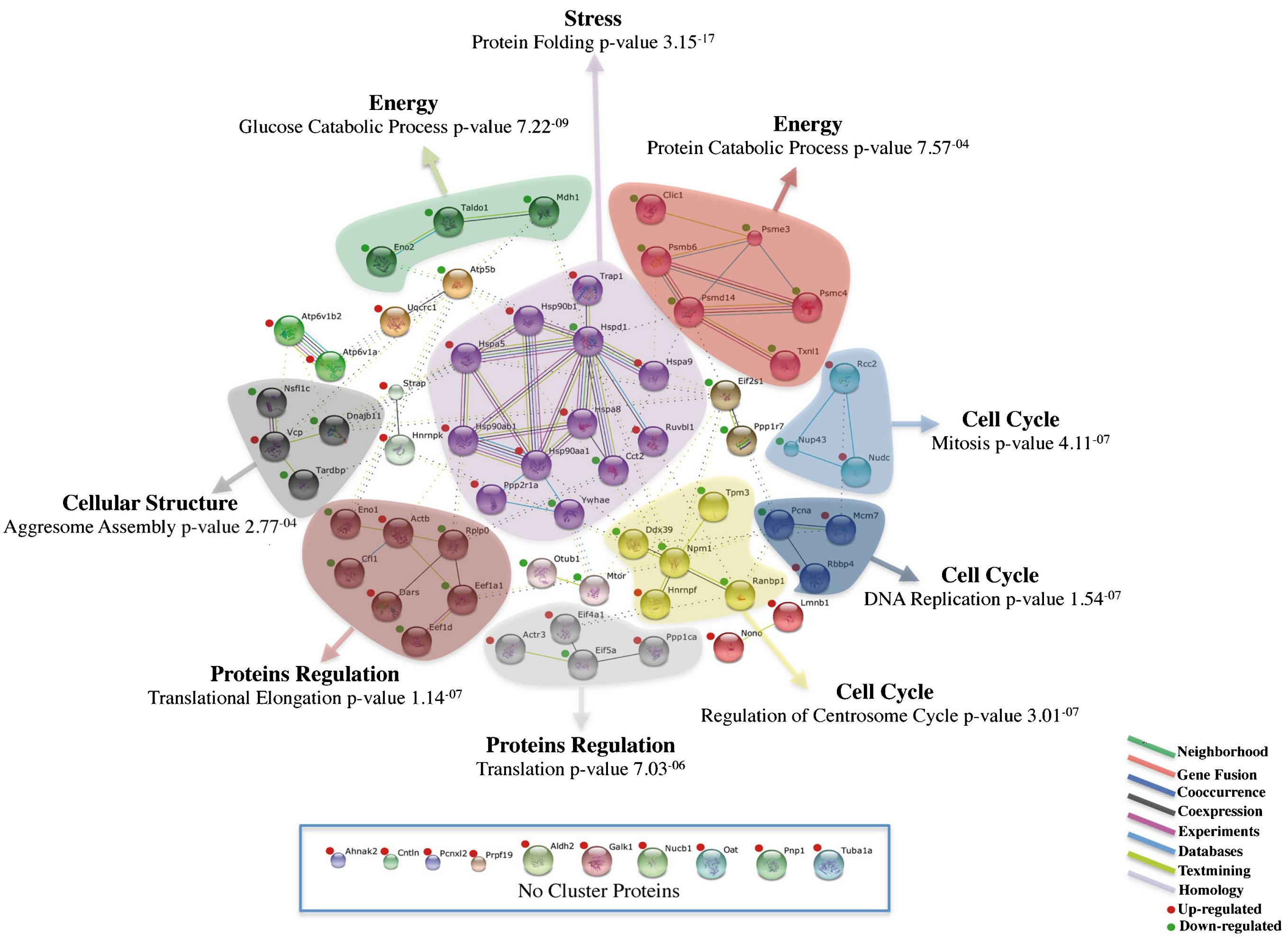

Fig. S5. Protein network of proteins identified in DIGE. Small red and green filled dots are representative of down- and upregulation, respectively. GO analysis of Cluster was performed with string software.

Table S1. List of the modulators used in screening, with the respective final concentrations.

Drug name	Concentration 1	Concentration 2	Concentration 3	Concentration 4
SAHA	5.0 µM	2.0 µM	1.0 µM	0.1 µM
VPA	10.0 µM	2.0 µM	1.0 µM	0.1 µM
Sodium butyrate	10.0 mM	1.0 mM	0.1 mM	0.01 mM
SK7068	5.0 µM	1.0 µM	0.5 µM	0.05 µM
Psammaplin A	5.0 µM	2.0 µM	1.0 µM	0.1 µM
MS-275	5.0 µM	2.0 µM	1.0 µM	0.1 µM
SB429201	5.0 µM	1.0 µM	0.5 µM	0.05 µM
Methylgene	5.0 µM	1.0 µM	0.5 µM	0.05 µM
SB379278	5.0 µM	1.0 µM	0.5 µM	0.05 µM
Sirtinol	100.0 µM	10.0 µM	1.0 µM	0.1 µM
EX527	500.0 nM	50.0 nM	1.0 nM	0.1 nM
BIX01294	10.0 µM	5.0 µM	1.0 µM	0.1 µM
ATRA	5.0 µM	1.0 µM	0.1 µM	0.01 µM
BMS641	10.0 µM	5.0 µM	1.0 µM	0.1 µM

Biology Open

Table S2. Mass spectrometry-identified hits involved in neural differentiation processes upon MS-275 treatment.

SPOT	Variation	Identification	Rules
536	30%	HSP90AB1	Potential role of heat shock proteins in neural differentiation of murine embryonic carcinoma stem cells
737	36%	VCP	Functional ATPase activity of p97/valosin-containing protein (VCP) is required for the quality control of endoplasmic reticulum in neuronal differentiated mammalian PC12 cells
904	52%	MCM7	Accumulation and dynamics of proteins of the MCM family during mouse oogenesis and the first embryonic cell cycle.
1028	27%	HSPA5	Oocyte-targeted deletion reveals that hsp90b1 is needed for the completion of first mitosis in mouse zygotes
1098	22%	HSPA9	Nuclear GRP75 binds retinoic acid receptors to promote neuronal differentiation of neuroblastoma
1448	55%	DARS	The signaling adapter protein DAP12 regulates multi-nucleation during osteoclast development
1513	21%	RUVBL1	Evolutionarily conserved transcriptional co-expression guiding embryonic stem cell differentiation (WNT pathway)
1758	46%	TUBA1A	Regulated expression of transgenes in embryonic stem cell-derived neural cells
1950	33%	NUDC	NudC is required for interkinetic nuclear migration and neuronal migration during neocortical development
2031	41%	AHNAK2	Self-regulated alternative splicing at the AHNAK locus

Table S3. Statistical analysis of mouse embryo chimera formation.

Cells treated with vehicle were injected in the 30 blastocysts
Cells treated with 0.5 µM of Entinostat were injected in the 30 blastocysts
Cells treated with 5.0 µM of Entinostat were injected in the 19 blastocysts

Readout and analysis:
10 embryos pretreated *in vitro* with vehicle
10 embryos pretreated *in vitro* with 0.5 µM of Entinostat
9 embryos pretreated *in vitro* with 5.0 µM of Entinostat

EGFP evaluation:
8 out of 10 embryos (vehicle) showed broad EGFP signal
7 out of 10 embryos (0.5 µM MS-275) showed broad EGFP signal
All 9 embryos (5.0 µM MS-275) showed a clear absence of EGFP signal
